# Simulated and natural changes in intraocular transparency selectively affect photoreceptor-specific pupil responses

**DOI:** 10.1038/s41598-026-54502-3

**Published:** 2026-05-29

**Authors:** María Constanza Tripolone, Roberto Sánchez, Luis Alberto Issolio, Pablo Alejandro Barrionuevo

**Affiliations:** 1 Instituto de Investigación en Luz, Ambiente y Visión (ILAV), CONICET - UNT, San Miguel de Tucumán, Argentina; 2 Complejo Astronómico “El Leoncito” (CASLEO), CONICET – UNC – UNLP – UNSJ, San Juan, Argentina; 3 Departamento de Luminotecnia, Luz y Visión, Facultad de Ciencias Exactas y Tecnología, UNT, San Miguel de Tucumán, Argentina; 4 Sensorimotor Learning Unit, Department of Psychology, University of Marburg, Marburg, Germany

**Keywords:** Neuroscience, Psychology, Psychology

## Abstract

The pupillary light reflex (PLR) is modulated by retinal photoreceptors and influenced by light transmission through the intraocular media (IOM). Variations in IOM transparency, due to scattering, transmittance, or yellowing, can affect visual and non-visual functions; however, their impact on photoreceptor-specific PLR remains unclear. In this study, we measured PLR responses selectively driven by Melanopsin, S-cones, and L+M cones in young adults using silent substitution and chromatic pupillometry. These pupillary measurements were correlated with individual IOM properties. Additionally, we simulated changes with external filters. Our findings indicate that the degree of scattering or transmittance impacts the cone-driven responses. Notably, increased scattering consistently slowed S-cone-driven responses across both experiments. Melanopsin-driven PLR and yellowing showed no significant effects. These results suggest that normal variations in IOM transparency modulate cone-mediated PLR dynamics but do not affect melanopsin responses, thereby contributing to individual differences in pupil behavior and potentially influencing ophthalmic and neurological assessments that rely on optical stimuli.

**Supplementary Information:**

The online version contains supplementary material available at 10.1038/s41598-026-54502-3.

## Introduction

When an increase in light intensity enters the eye, the pupil constricts; this behavior is called the pupillary light reflex (PLR). The PLR is driven by retinal photoreceptors via brain centers such as the Pretectal Olivary nucleus and the Edinger-Westphal nucleus^1^. However, before reaching the retina, light must first pass through the intraocular media (IOM). Therefore, the transparency of the IOM is expected to play an important role in the PLR.

The transparency of the IOM can be affected by variations in transmittance, scattering, and color^2^. Striking changes in these properties can result from ophthalmic diseases such as cataracts^3,4^, aging^5^, or even corneal implants^6^. These alterations can affect the pupillary response; for example, it has been shown that PLR amplitude is reduced with age as the lens becomes more yellow^7–9^.

However, even within a normal young population, there is considerable variability in transmittance and intraocular scattering values. Van Norren and Vosestimated that individual differences among young observers (without age-related lens yellowing) vary by up to 25% from the average value^10^. For visualpurposes, these differences can largely be compensated by light^11,12^, color^13^, and scattering adaptation^14^. In contrast, non-visual functions respond to thelevel of irradiance. Indeed, it has been shown that melatonin suppression is affected by lens transmittance in a normal healthy population^15^. Since the PLR isalso a non-visual function that reacts to optical radiation, differences in ocular media transparency within a normal cohort might be related to changes in thePLR. Although it is assumed that this normal variability in ocular transparency is small enough to impact the pupil light reflex, to our knowledge, this hypothesishas not yet been tested.

Each type of photoreceptor contributes differently to the photopic PLR in terms of their nature and activation^16^. While cones are the main drivers of the early transient pupillary response^17,18^, melanopsin activation helps maintain the pupil constriction even after the stimulus is turned off^19,20^. The contribution of melanopsin and L+M cones (luminance) is excitatory, whereas S-cones contribution has an inhibitory behavior^21,22^. However, a pulse of S-cones stimulation also produces an initial pupil contriction^23,24^. Furthermore, the activation threshold of melanopsin is higher than that of cones and rods^25,26^. Anatomical characteristics also contribute to this photoreceptor differentiation. In the human retina, cones are highly concentrated in the fovea region, decreasing gradually towards the periphery^27^, whereas melanopsin-expressing intrinsically photosensitive retinal ganglion cells (ipRGCs) are absent in the fovea pit and reach the highest concentration in the parafovea^28,29^. Since the spatial distribution of ipRGCs is different from that of cones, a variation in intraocular scattering is expected to differentially affect the PLR driven exclusively by one photoreceptor type or by a single postreceptoral pathway.

Large changes in scattering and transmittance occur naturally with age; however, aging also affects the PLR due to oculomotor factors such as senile miosis^30,31^. Therefore, to study the effects of changes in intraocular transparency, it is preferable to consider a group of young participants, thereby minimizing the influence of age-related changes in pupil motility. Although visual optics and pupillometry are both well-established research fields, the physiological relationship between intraocular media changes and pupillary dynamics has been scarcely studied. This work aimed to determine the impact of variation in scattering, transmittance, and yellowing of the intraocular media on photoreceptor-specific pupil light reflex.

## Results

### Responses to selective stimulation

#### Flicker paradigm

**Fig. 1:**
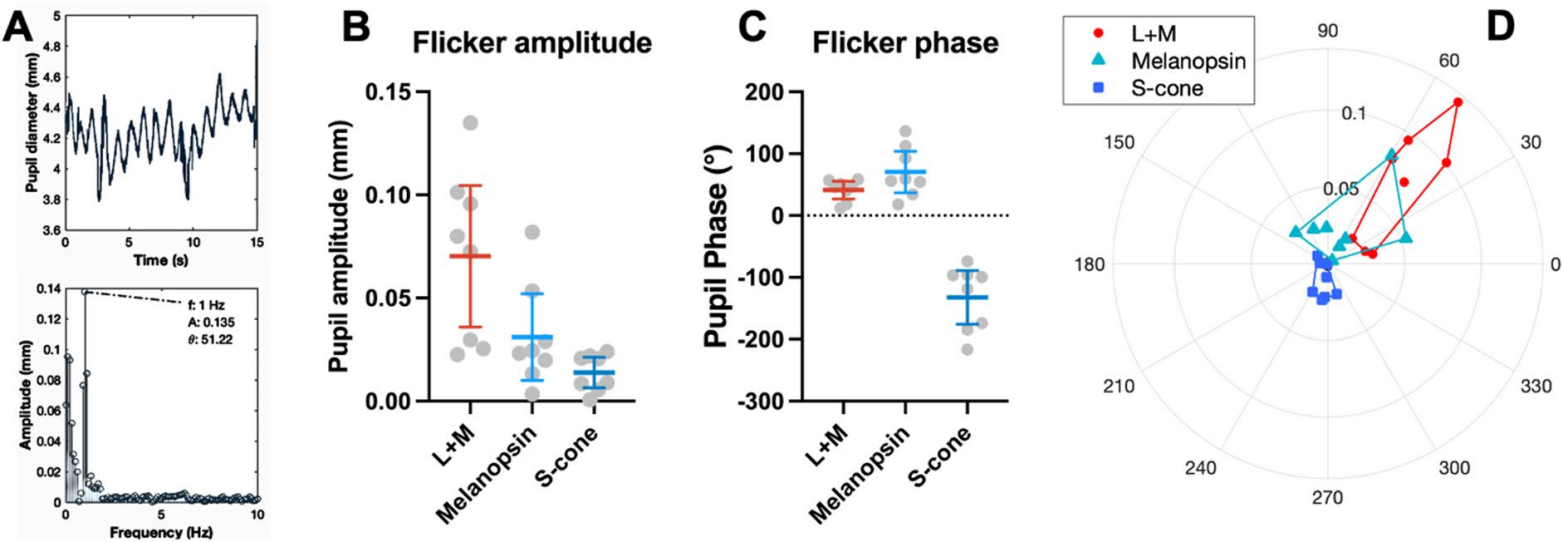
Pupillary responses to the flickering stimulation of Melanopsin, S-cones, and L+M cones. (**A**) One participant pupil diameter in the time domain (upper panel) and its amplitude in the frequency domain (lower Panel) for the L+M cones condition (note the highlighted amplitude at the fundamental frequency of 1 Hz). (**B**) Flicker amplitude and (**C**) Flicker phase box plots for the three photoreceptor conditions (grey dots are the individual’s average values). (**D**) Polar plot representing the individual pupil amplitude and phase for the L+M cones (red), Melanopsin (cyan), and S-cones (blue). Error bars represent the confidence interval.

Selective stimulation of individual or combined photoreceptors classes was achieved implementing the silent substitution technique^32^ (see Methods section). With this approach, we measured the pupil response to a flickering stimulus (fPLR) for three photoreceptor conditions: Melanopsin, S-cones, and L+M cones (luminance). Figure 1 shows the amplitude and phase responses at the fundamental frequency (1 Hz). As expected, the higher pupil amplitudes were observed for the L+M stimulus (Figs. 1A-B). Melanopsin response was smaller and exhibited a slightly different phase. S-cones responses showed the smallest pupil amplitudes (Fig. 1B), and were out of phase with both Melanopsin and L+M (Figs. 1C-D), which is a signature of the ipRGC system in humans^25^. These results are consistent with previously reported pupillary results^21,22,24,33,34^.

#### Pulsed paradigm

**Fig. 2:**
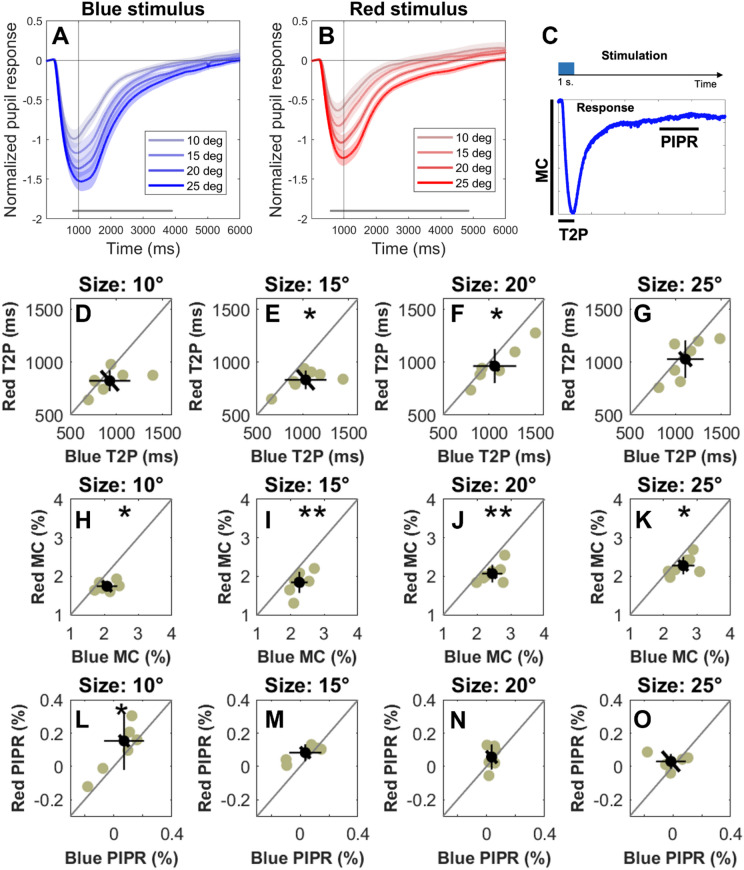
Pupillary responses to blue and red pulse stimulation for stimulus sizes of 10, 15, 20, and 25 degrees. (**A**) Blue stimulus and (**B**) Red stimulus mean pupil traces (solid lines) for the four stimulus sizes (shadow areas indicate the standard error of the mean for each trace). The gray thin vertical lines represent the offset of the stimulation, and the wide gray horizontal lines show the section where the curves are significantly different. (**C**) Pupillary response to a 1-s pulse stimulus showing the three parameters: time to constriction peak (T2P), the maximum constriction (MC), and the post-illumination pupil response (PIPR). (**D**–**O**) Pupillary parameters of red versus blue pulse, showing the participants’ values (grey dots), the mean values (black dots), the stand-alone effect confidence interval (vertical and horizontal thin black lines), and the confidence interval for the pairwise differences (diagonal wide black lines). The T2P (**D**–**G**), the MC (**H**–**K**), and the PIPR (**L**–**O**) parameters were plotted for stimulus sizes from 10° to 25° (left to right panels, respectively). (*) *p* < 0.05, (**) *p* < 0.01.

Another way to assess the effect of different photoreceptors in the PLR is through chromatic pupillometry^35,36^ (see Methods section). Using this technique, we measured the pupillary response to a pulse (pPLR) of blue (Fig. 2A) or red (Fig. 2B) stimulation for four stimulus sizes. According to the literature, the red-driven phasic part of the pPLR is mainly driven by L+M cones^17^, whereas blue-driven phasic component is primarily driven by S-cones and rods^37^, and the blue-driven tonic response after stimulation is mostly mediated by melanopsin^20^. Therefore, we calculated three parameters (Fig. 2C): The time to constriction peak (T2P), the maximum constriction (MC), and the post-illumination pupil response (PIPR). Analysis of general effects showed that the MC was significantly higher for a stimulus size of 25° compared to 10° (T = 5.04; *p* < 0.001) and 15° (T = 3.60; *p* < 0.05). This analysis also revealed that the blue MC was higher than the red MC (T = 4.58; *p* < 0.001). In pairwise comparisons between red and blue stimulation, we found that both the MC and T2P were higher for blue stimuli than for red (Fig. 2D–K). Significant T2P differences were observed at two stimulus sizes (15°: t = 2.6, *p* = 0.04; 20°: t = 2.57, *p* = 0.042). MC differences were significant at all four sizes (10°: t = 3.07, *p* = 0.028; 15°: t = 4.51, *p* = 0.004; 20°: t = 3.77, *p* = 0.009; 25°: t = 2.74, *p* = 0.034). When analyzing the PIPR, the pupil diameter was larger for red stimuli compared to blue (Fig. 2L–O,), significantly at the smallest size (t = − 2.93, *p* = 0.026). These results are consistent with the typical results of chromatic pupillometry in healthy individuals^20,38–40^.

To evaluate the effects of IOM transparency on the photoreceptor-specific pupil response in a young normal group, we conducted two experiments. In the first, the relation experiment, we assessed the associations between the IOM transparency variables (scattering and transmittance) with the fPLR variables. In the second, the simulation experiment, we simulated changes in the IOM variables using external filters (Fig. S1) and analyzed the effect of IOM scattering, transmittance, and yellowing on the pPLR.

### Scattering effect

#### Relation experiment

**Fig. 3:**
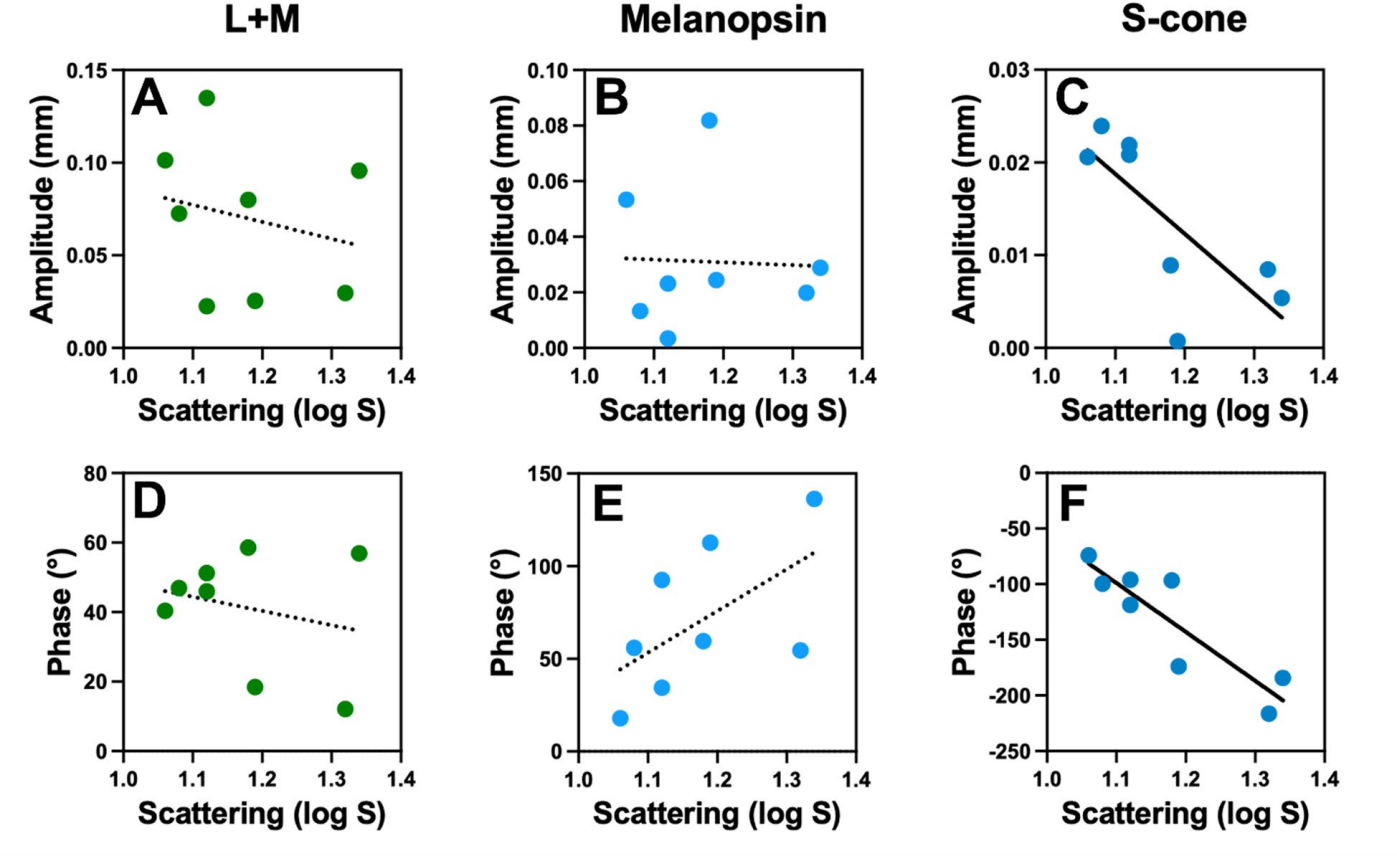
Correlations between flickering pupillary responses and intraocular scattering in the relation experiment. Amplitude (upper panels) and phase (lower panels) versus scattering parameter (log S) plotted for the L+M cones (**A** and **D**), Melanopsin (**B** and **E**), and S-cone (**C** and **F**) photoreceptor conditions (dots represent the participants’ values). The S-cone responses were significantly associated with the log S (solid lines), while the remaining correlations did not reach statistical significance (dotted lines).

When analyzing the correlation between intraocular scattering with fPLR variables, we didn’t find an association with the L+M and Melanopsin variables (Fig. 3A, B, D, E). However, the scattering level was significantly correlated with S-cone amplitude (Fig. 3 C; R^2^ = 0.574, *p* = 0.029) and phase (Fig. 3 F; R^2^ = 0.792, *p* = 0.003). This indicates that, as the scattering increases, the pupil size variation is smaller and the pupillary reflex is slower for S-cones.

#### Simulation experiment

**Fig. 4:**
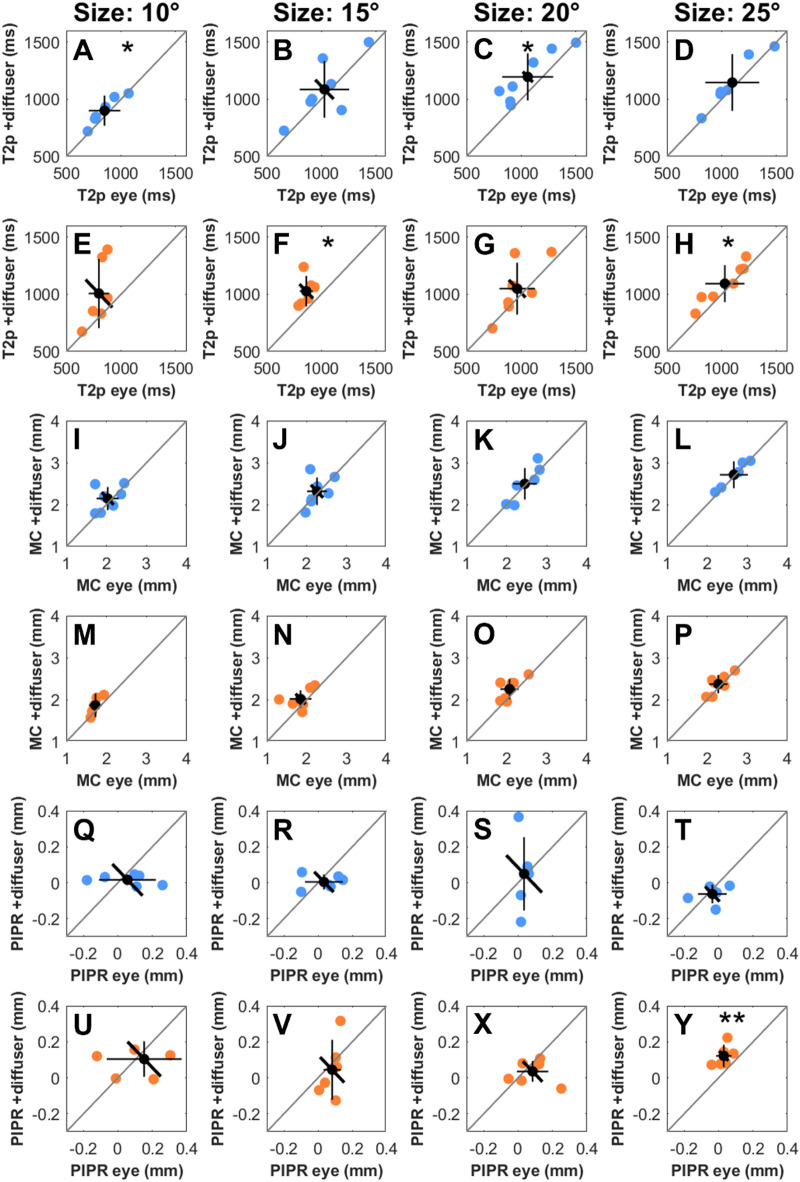
Effect of an external scattering filter on the pupillary responses to pulse stimulation in the simulation experiment. (**A**–**Y**) Pupillary parameters using a diffuser filter versus naked eye (without filter) for stimulus sizes from 10° to 25° (arranged in columns from left to right), showing the mean values (black dots), the stand-alone effect confidence interval (vertical and horizontal thin black lines), and the confidence interval for the pairwise differences (diagonal wide black lines). The blue and red dots represent the participants’ values for the blue and the red pulses, respectively. (**A**–**H**) Time to constriction peak (T2P). (**I**–**P**) Maximum constriction (MC). (**Q**–**Y**) Post-illumination pupil response (PIPR). [(*) *p* < 0.05, (**) *p* < 0.01].

To confirm the relationship between the scattering and the PLR, we conducted a second experiment using pulsed stimulation and an external diffusion filter to simulate a significant increment in scattering^41–43^. We also tested different stimulus sizes to check if the effect of scattering depends on the amount of light flux. The results of this experiment are shown in Fig. 4. We found that using the scattering filter, the time to constriction peak for both the blue and red pPLR increased (Fig. 4A-H). This increment was significantly for 10° (t = -3.138, *p* = 0.026) and 20° (t = -3.612, *p* = 0.011) of blue stimulation, and for 15° (t = − 3.369, *p* = 0.02) and 25° (t = − 3.016, *p* = 0.024) of red stimulation. This means that, as light diffusion increases, the pupillary response is slower. Therefore, with an external diffuser, we could confidently replicate the S-cones findings from the flicker paradigm of the relation experiment. We didn’t find any significant effect of the scattering filter for the maximum constriction at any color or size in pairwise comparison (Fig. 4I–P). When we assessed the effect of the size and color, we found that a post-hoc analysis of a repeated-measures ANOVA indicated a significantly lower MC for stimulus size of 10° than for 20° (T = 3.59; *p* < 0.05) and 25° (T = 5.02; *p* < 0.001). Without filter we found a similar effect when comparing 10° and 25° (T = 5.040; *p* < 0.0002). The blue MC was found higher than the red (T = 3.93; *p* < 0.01) in agreement with the results without filter (T = 4.577; *p* < 0.0002).

Regarding the PIPR, the scattering filter caused an increment in this pPLR variable only for the red stimulus at the largest stimulus size (Fig. 4Y; t = − 4.421, *p* = 0.007). However, this effect was not translated to the difference between the blue PIPR and the red PIPR (Fig. S2), which is a metric used to assess melanopsin effect^44,45^.

### Transmittance effect

#### Relation experiment

**Fig. 5:**
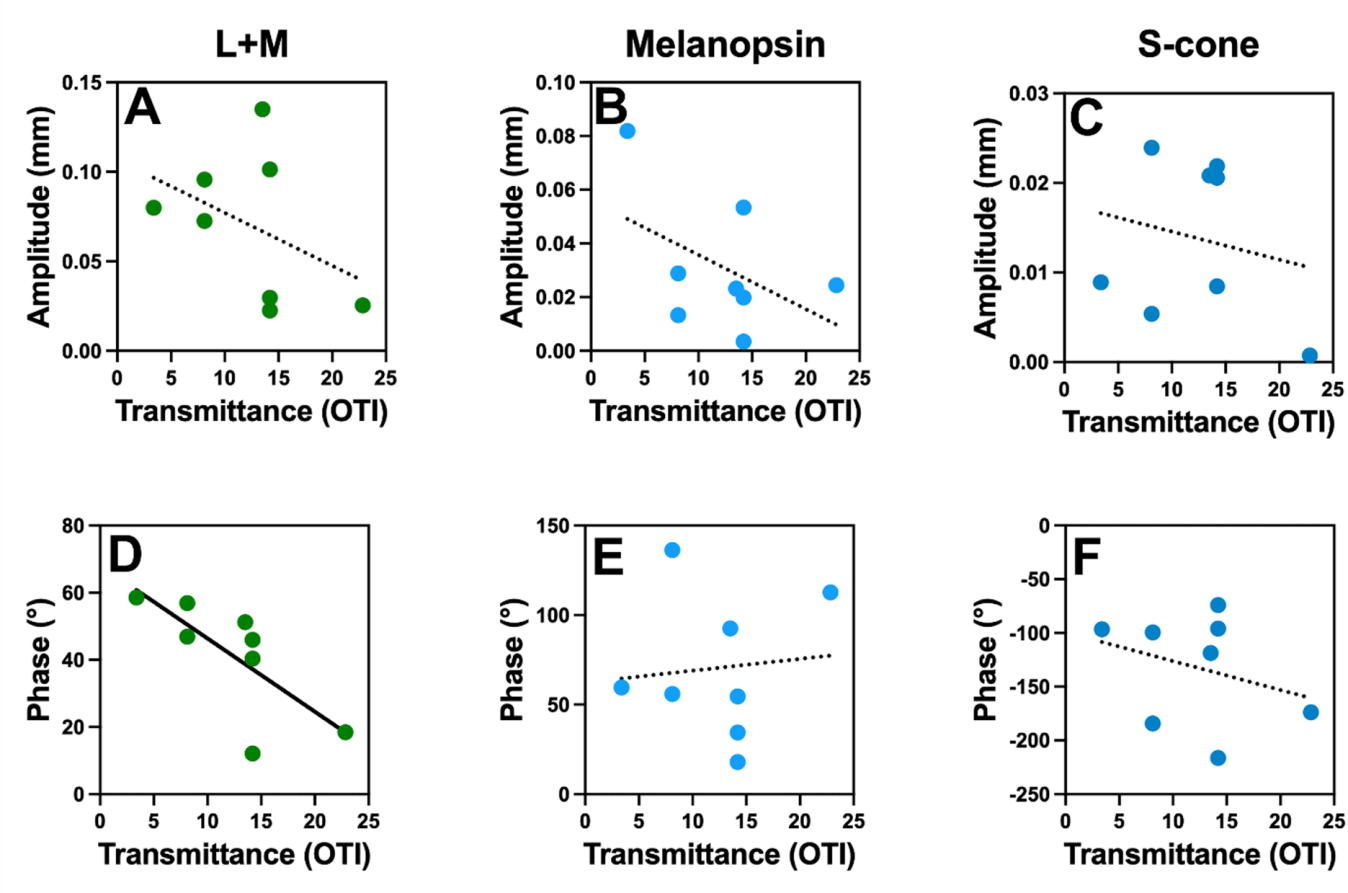
Correlations between flickering pupillary responses and intraocular transmittance in the relation experiment. Amplitude (upper panels) and phase (lower panels) versus ocular transmittance index (OTI) plotted for the L+M cones (**A** and **D**), Melanopsin (**B** and **E**), and S-cone (**C** and **F**) photoreceptor conditions (dots represent the participants’ values). The L+M cones pupillary phase was significantly associated with the OTI (solid line), while the remaining correlations did not reach statistical significance (dotted lines).

When analyzing the correlation between fPLR variables with intraocular transmittance, no significant associations were found for the amplitude of the L+M (Fig. 5A), Melanopsin (Fig. 5B), and S-cones (Fig. 5C) conditions. In contrast, the L+M phase was negatively correlated with the ocular transmittance index (OTI) (Fig. 5D; R^2^ = 0.549, *p* = 0.036). This means that, as the transmittance increases, the PLR is slower for the luminance condition. The remaining fPLR phase variables were not correlated with the transmittance level (Fig. 5E–F).

#### Simulation experiment

**Fig. 6:**
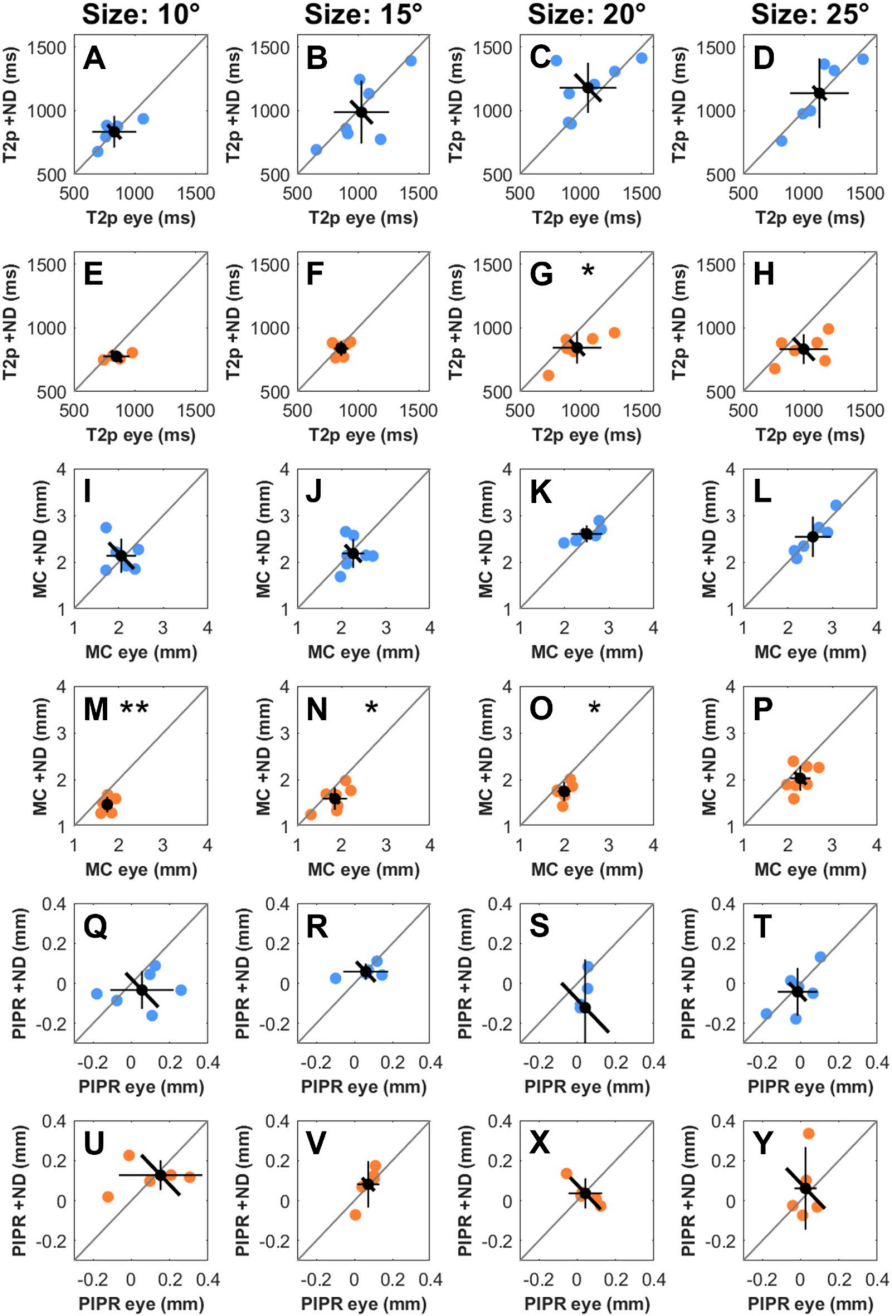
Effect of an external neutral density filter on the pupillary responses to pulse stimulation in the simulation experiment. (**A**–**Y**) Pupillary parameters using a neutral density filter (ND) versus naked eye (without filter) for stimulus sizes from 10° to 25° (arranged in columns from left to right), showing the mean values (black dots), the stand-alone effect confidence interval (vertical and horizontal thin black lines), and the confidence interval for the pairwise differences (diagonal wide black lines). The blue and red dots represent the participants’ values to the blue and the red pulses, respectively. (**A**–**H**) Time to constriction peak (T2P). (**I**–**P**) Maximum constriction (MC). (**Q**–**Y**) Post-illumination pupil response (PIPR). (*) *p* < 0.05, (**) *p* < 0.01.

To further study the effect of a change in transmittance in the pPLR, we used an external foiled neutral density filter (64% of transmittance) to simulate an opacity increase. We also tested different stimulus sizes to check if the effect depends on the amount of light flux (Fig. 6). When reducing transmittance by an external neutral density filter, we found that the time to constriction peak for the red stimulus was shorter in comparison with the naked eye, especially for 20° (Fig. 6G; t = 2.632, *p* = 0.046). This indicates that the PLR is slower for higher transmittance, in agreement with L+M cones phase results in the relation experiment. We also found a notable reduction in the maximum constriction to the red stimulus for all sizes, which was significant for 10° (Fig. 6M; t = 4.07, *p* = 0.01), 15° (Fig. 6N; t = 2.95, *p* = 0.026), and 20° (Fig. 6O; t = 3.321, *p* = 0.021). This means that a lower light availability produces a reduction in the strength of the PLR, as expected. Regarding the other pupillary variables, we didn’t find differences between the naked eye and the eye with filter.

When we assessed the effect of the size and color, we found a significant increase in MC for stimulus size of 25° compared to 10° (T = 4.47; *p* < 0.01) and 15° (T = 3.66; *p* < 0.05). In addition, the blue pupil results were higher than the red for the T2P (T = 3.69; *p* < 0.01) and the MC (T = 8.49; *p* < 0.001) variables. Comparing with the scattering condition, the pupil results to red stimulus color of the transmittance filter were lower than those for the scattering filter (T2P: T = 3.89; *p* < 0.01. MC: T = 5.40; *p* < 0.001). Finally, we found that the red MC of the naked eye was increased from that of the transmittance filter (T = 3.72; *p* < 0.01).

### Yellowing effect

#### Simulation experiment

**Fig. 7:**
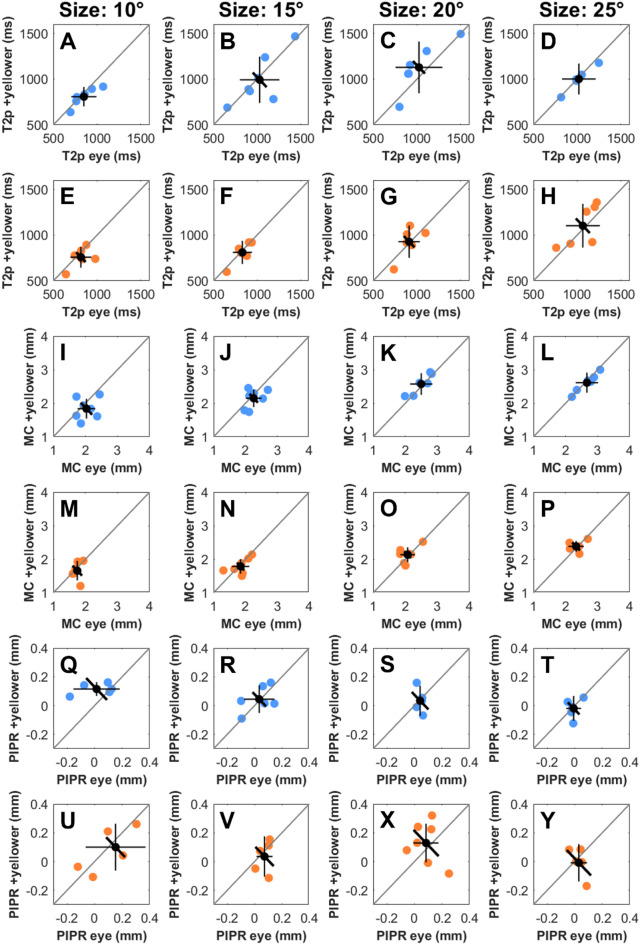
Effect of an external yellow density filter on the pupillary responses to pulse stimulation in the simulation experiment. (**A**–**Y**) Pupillary parameters using a yellow filter (yellower) versus naked eye (without filter) for stimulus sizes from 10° to 25° (arranged in columns from left to right), showing the mean values (black dots), the stand-alone effect confidence interval (vertical and horizontal thin black lines), and the confidence interval for the pairwise differences (diagonal wide black lines). The blue and red dots represent the participants’ values to the blue and the red pulses, respectively. (**A**–**H**) Time to constriction peak (T2P). (**I**–**P**) Maximum constriction (MC). (**Q**–**Y**) Post-illumination pupil response (PIPR). No significant differences were found in this filter condition.

To further study the effect of a color change, we used an external yellow filter to simulate the lens yellowing effect (Fig. 7). We also tested different stimulus sizes to check if the effect depends on the amount of light flux. When changing the spectral transmittance by an external yellow filter, we didn’t find any difference between the pupillary variables for the naked eye and the filter interposed condition.

When we assessed the effect of the size and color, we found that the MC was significantly lower for the stimulus size of 10° than 20° (T = 5.72; *p* < 0.001) and 25° (T = 6.91; *p* < 0.001). Similarly, the MC for 15° was decreased compared with 20° (T = 3.58; *p* < 0.05) and 25° (T = 4.80; *p* < 0.001). Regarding the stimulus color, the blue pupil MC was significantly higher than the red MC (T = 3.99; *p* < 0.01). In addition, the red MC for the yellowing filter was significantly higher than that for the transmittance filter (T = 7.80; *p* < 0.01).

### Overall simulation effect

We also tested the overall changes introduced by the manipulations in the simulation experiment. We performed a repeated-measures ANOVA to assess the effects of stimulus color (blue or red), filter (no filter, diffuser, ND, and yellower), and size (10° to 25°) in the T2P, MC, and PIPR variables. The results showed significant effects of color (F = 16.42; *p* < 0.001), filter (F = 4.71; *p* < 0.01), and size (F = 11.02; *p* < 0.001) in the T2P. For the pupil MC, the effects of color (F = 110.79; *p* < 0.001), filter (F = 6.07; *p* < 0.01), and size (F = 46.87; *p* < 0.001) were significant, as well as the interaction of color and filter (F = 4.96; *p* < 0.01). For the PIPR, we found significant effects of stimulus color (F = 15.41; *p* < 0.001) and size (F = 4.04; *p* < 0.01), but no effect of the filter. These results suggest that cones, but not melanopsin are affected by the insertion of any of the external filter that were used in this study.

## Discussion

**Fig. 8:**
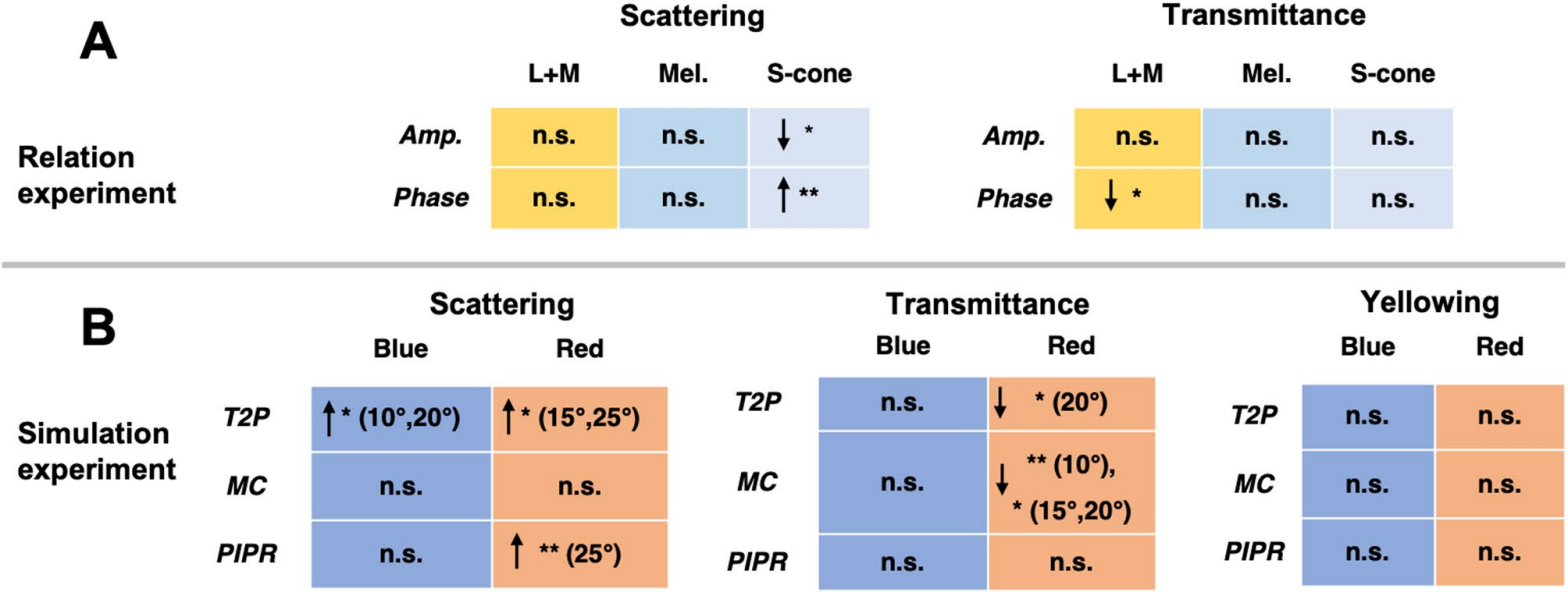
Schematic summary of the significant effects of intraocular media transparency on the photoreceptor-specific pupillary light reflex. (**A**) Relation experiment: significant correlations between fPLR variables (amplitude and phase at 1 Hz) and intraocular scattering (log S) and transmittance (OTI) for the L+M, melanopsin (Mel.), and S-cone photoreceptor conditions. Arrows indicate positive (↑) or negative (↓) correlation. (**B**) Simulation experiment: significant pairwise differences between naked eye and the diffuser, neutral density (ND), and yellow filter conditions, for the time to constriction peak (T2P), maximum constriction (MC), and post-illumination pupil response (PIPR), separated by stimulus color (blue or red) and indicating the stimulus sizes at which significance was reached. Arrows indicate the direction of the effect (↑ increase, ↓ decrease relative to the naked eye). n.s.: not significant; *p < 0.05; **p < 0.01.

In this work, we assessed how photoreceptor-specific pupillary responses are influenced by variations in the intraocular media transparency. Using two pupillometric paradigms to selectively stimulate specific photoreceptor types, we found that the level of scattering or transmittance affected the cone-driven but not the melanopsin-driven pupil responses. Instead, we didn’t find a significant effect of the yellowing lens variation in the PLR. These effects were consistent across different devices and approaches, analyzing the relationship between variables and simulating changes in the pre-receptoral media. To facilitate an overview of the pattern of significant effects across photoreceptor inputs, experimental paradigms, and IOM manipulations, we summarize the main results in Fig. 8.

In our first experiment, we found that a small increment in intraocular scattering made the S-cone-driven pupillary light reflex slower (Fig. 3F). The simulation experiment confirmed that an increment in scattering produced sluggish blue-driven pupil response (Fig. 4). This constitutes the main finding of this study. Since rod stimulation was steady because we used the silent substitution in the flickering paradigm, the scattering effect seems to be produced mainly by S-cones in the first experiment. However, for the simulation experiment, we cannot discard a potential contribution of rods. Physically, an increment in intraocular scattering generates a spread of the stimulation area while maintaining the light flux density. This area increment means that more photoreceptors are reached by the stimulation. However, it is not clear how this increment in stimulation area delays the pupil response. Only a few studies dealt with stimulus area and temporal parameters of the pPLR transient component. It was found that an increase in the stimulation area reduces the PLR latency^46^, which does not agree with our findings. However, this previous paper dealt with white light; instead, we used chromatic light. From another study that analyzed the role of size area in chromatic pupillometry, we can infer that the time to peak of the blue PLR is slower for higher stimulus area in dark-adapted conditions, but this trend disappeared with higher stimulus contrast and for light-adapted conditions^40^. Therefore, a change in this timing might be related to rod signals. In this way, we cannot rule out the contribution of rods, especially in the simulation experiment.

Van Norren and Vos showed that most of the individual differences in young observers are particularly produced for wavelengths below 420 nm^10^. Therefore, these intraocular scattering individual differences might be mainly high in the shortwave range. This effect can be explained by the scattering nature of the intraocular media, which scatter most at the short wavelengths^47^. Especially for small particle sizes where the Rayleigh method can be applied^48^. However, this effect is not a property of the external filter included in the simulation experiment. Although our filter mimics several characteristics of an early cataract^41,42,49^, it was shown that this filter does not mimic the wavelength dependency of the intraocular lens^50^. This might explain the small or non-significant effect found with the filter, considering different stimulation sizes.

Interestingly, a higher level in IOM transmittance produced a slower pupil response according to the L+M phase results (Fig. 5D). This effect was confirmed in our simulation experiment for the red stimulus. The time to constriction peak was slower for the naked eye than for the eye with the neutral density filter. This effect is significant for the 20-degree stimulus size (Fig. 6G). This result was not expected since previous studies showed that the latency is shorter and the peak velocity is higher as the stimulus size increases^51–55^. However, latency and peak velocity (usually computed as first derivative) may not reflect the time to constriction peak, as we showed in the supplementary material (Fig. S3). Additionally, we found a consistent reduction in the MC by wearing the neutral density filter (Fig. 6M–P). This result was expected due to a reduction in light intensity. However, this effect was not found in the relation experiment (Fig. 5), which might be related to the fact that the changes in natural transmittance are smaller than those produced by the ND filter, and foveal cone stimulation was left aside by the use of the central dark disk (see the Methods section).

In the relation experiment, only the L+M-cone temporal response was associated with natural transmittance; however, the temporal response of pupil typically becomes slower at lower retinal illumination across all photoreceptor classes^18^. A possible explanation is that the L+M signal is the dominant input to the photopic flicker PLR and produced the largest response in our paradigm (Fig. 1B). Smaller flicker responses, such as those of S-cones and melanopsin, may be less sensitive to detect modest changes in retinal illuminance such as those produced by natural transmittance variability in young observers. The simulation experiment, which used a stronger reduction in transmittance, did show effects on the red MC and T2P, in agreement with the expected slowing and reduction of the cone-mediated response at lower illuminance.

From our two experiments, none of the melanopsin-driven PLR parameters were related to scattering, transmittance, nor yellowing level. Therefore, the melanopsin system is insensitive to variation in transparency of a young group and even simulating important changes in transparency that mimic eyes from an older group. A previous study assessing melatonin suppression showed differences when comparing children and adults, in disagreement with our results^15^. This discrepancy might be related to the fact that we didn’t test children who have a more transparent media than adults^2,56,57^. Our finding suggests the robustness of the melanopsin-driven pupil response in adults for signaling large changes in light exposure^58^.

Our findings have implications for the clinical use of chromatic pupillometry. The lack of a significant effect of the yellowing filter and the resilience of melanopsin-driven responses across our manipulations are consistent with previous studies of healthy aging showing that lens yellowing has a negligible effect on pupil responses to short-wavelength light^8,59^, although other reports describe age-related enhancements in melanopsin-driven pupil responses^60^. The robustness of melanopsin signaling is particularly relevant for clinical applications in conditions where the lens ages prematurely, such as diabetes^61,62^, where ocular media changes may be exacerbated. As an alternative for elderly or aphakic patients, chromatic pupillometry protocols using a green light in place of the blue light have been proposed to negate the effect of lens yellowing on the melanopsin PIPR^63^. In line with previous work, we also computed the net PIPR (the difference between the blue PIPR and the red PIPR), which has been used as a metric of melanopsin contribution in glaucoma^64,65^, and found no effect of the scattering filter on this metric (Fig. S2). Together, these results support the use of melanopsin-targeting pupillometry protocols as biomarkers of inner-retinal function relatively unaffected by typical IOM variations in young adults.

We implemented two techniques that constitute the gold standard for selectively stimulating melanopsin, S-cones, and L+M cones. These are the silent substitution method and the chromatic pupillometry. Regarding the silent substitution results, this technique was implemented in conjunction with sinusoidal stimulation. Our results are consistent with previous studies that employ a comparable paradigm, regardless of whether they used similar^22,33^ and different^21^ apparatus. In particular, we confirmed the S-cone out of phase as well as the similar phase between melanopsin and L+M cones, which was previously reported. Although the amplitude depends on the implemented contrast, our results showed that S-cones and melanopsin have a much smaller response than L+M stimulation, even though they have higher (S-cones) or similar (melanopsin) contrasts than L+M stimulation, in agreement with previous studies using 50% of contrasts^21^ and a similar contrast range^22^. Regarding chromatic pupillometry, we followed the protocol advised by previous authors to assess the contributions of melanopsin, rods, and cones in the PLR^20,24,39^. As expected for the phasic part, mainly driven by cones, the blue constriction response was larger and slower than the red response (Fig. 2A), in agreement with previous data^39,66^. For the PIPR, mediated by melanopsin, the blue stimulation produced a higher sustained response (smaller pupil size) than the red stimulation (Fig. 2L–O), in agreement with the literature^20,39^.

A potential concern with the implemented paradigms is that photoreceptor excitations will be affected by changes in transparency. In the relation experiment, these changes would affect the silent substitution computation. However, a heterochromatic flicker photometry (HFP) test was conducted to account for these small natural changes (See Methods). In the simulation experiment, the changes introduced by the filters will significantly modify photoreceptor excitations. We computed the excitation changes introduced by the filters (Fig. S1D, E, and F). The pattern in the change in excitations agrees only with the transmittance effect found for the red stimulus (Fig. 8B). However, the scattering effects did not follow the excitations changes. Therefore, this effect may be better explained by the interplay between the area reached by the scattering and the spatial distribution of photoreceptors.

A promising avenue for future research is to use the photoreceptor-directed approach itself to simulate the effects of IOM changes on the photoreceptor inputs to the pupil. Once the spectral filtering produced by yellowing, scattering, or transmittance changes has been characterized, the silent substitution method can be modified to reproduce the resulting changes in photoreceptor excitations directly, without the need for external filters. Specially important to simulate the scattering effect is to use a multi-primary device with possibility to generate spatially complex stimulation^67,68^. This approach would allow to isolate the specific transparency influence from any extra-photoreceptor optical effects (e.g., diffraction, glare, or contrast attenuation introduced by physical filters), thereby clarifying the mechanisms underlying our observations.

In this study, we found that small variation in intraocular transparency are associated with changes in the dynamics of photoreceptor-specific pupillary light responses. Our results indicate that melanopsin-driven responses are resistant to changes in scattering, transmittance, and yellowing of the intraocular media, whereas cone-driven responses, particularly those mediated by S-cones, are affected by variation in transparency. These findings suggest that part of the individual variability in the flickering and pulsed PLR of young observers can be explained by differences in intraocular media properties. Since the PLR depends on the light reaching the photoreceptors, the clarity of the intraocular media influences both the intensity and spatial structure of this light. Furthermore, variation in intraocular transparency may influence ophthalmic and neurological assessments that rely on optical stimuli.

### Limitations

While the sample size is modest in both experiments, the fact that the main findings were obtained in different cohorts and with different techniques suggests a consistent physiological effect. Also, given the novel nature of the role of IOM in the PLR, this study provides the physiological basis for the next large-scale studies to emerge. Furthermore, this sample size is common for silent substitution reports^23,69,70^ and for studies simulating changes in the intraocular media^41,49^.

The filters used in the simulation experiment were selected to mimic a natural shift in scattering, transmittance, and yellowing. However, they could not simulate a real change, as mentioned for the spectral characteristics of the scattering filter.

The groups of participants belong to different locations and ethnicities, which could make it difficult to compare the results. This is particularly important considering potential differences in iris pigmentation that can affect the intraocular scattering spectral profile^71^. However, there were only minimal differences in the distribution of iris color (Table S1). Indeed, it was shown that the PLR is not dependent on the iris pigmentation in a large natural exposure study^58^. Furthermore, this difference in ethnicities actually favors the generality of our main findings.

Beyond changes in light flux, manipulating the stimulus size also changes the relative contribution of different photoreceptor types and the corneal flux density of the stimulus. Cones are highly concentrated in the foveal region and decrease toward the periphery, rods reach maximum density at intermediate eccentricities, and ipRGCs are mostly concentrated around the parafovea, all of which leads to a varying mix of photoreceptor signals as the stimulus area expands. In our pulsed paradigm, foveal cone stimulation was excluded by a 5°-radius dark patch, so the annular stimuli (5°–25°) progressively recruited parafoveal and peripheral photoreceptors. While we cannot fully exclude an additional spatial effect of rod intrusion in the pulsed paradigm, the consistency of the scattering-induced delay in S-cone responses across both the flicker (where rod stimulation was silenced via silent substitution) and pulsed paradigms supports a true cone-mediated effect. Stimulus size also modifies the corneal flux density and thereby the absolute photoreceptor excitations, an effect that has previously been reported to influence chromatic pupil metrics^40,72^. The interpretation of the photoreceptor inputs to the phasic and tonic components of the PLR has also been discussed in detail elsewhere^19,73^.

## Methods

### Experiments

We conducted two experiments in this study. In the first one, a relation experiment, we analyzed the correlation between pre-receptoral variables and pupillary variables obtained in the same sample of young participants. In the second one, a simulation experiment, we simulated exacerbated pre-receptoral variables using external filters, and we measured the effect of these filters on the PLR of a second sample of young participants.

### Participants

#### Relation experiment

Eight young observers, four males and four females (age range: 25 to 38 y.o., mean: 31 ± 5 y.o.), with no ocular pathology, participated in the experiment. The observers were recruited from a university cohort, including students, laboratory coworkers, and two of the authors. All participants had normal visual acuity (range: -0.2 to 0.1 logMAR) (logMAR Visual Acuity Chart; Vector Vision, Greenville, USA), and normal color discrimination (Farnsworth-Munsell 100-Hue Test, X-Rite Pantone, USA), except one participant scored as having superior discrimination. The participants provided informed consent and were recruited in the city of San Miguel de Tucumán, Argentina, with Latin American origin. Informed consent was obtained from all participants before the experiment, and procedures adhered to the tenets of the Declaration of Helsinki. The study protocols were approved by the Research Ethics Committee of the National University of Tucumán (number 28/2018; approved on 4 September 2018.

#### Simulation experiment

Seven young observers, two males and five females (age range: 20 to 45 y.o., mean: 28.6 ± 9.5 y.o.), with no ocular pathology, participated in the study. All the participants in this experiment had normal or corrected to normal visual acuity, and normal color vision. The participants provided informed consent and were recruited in the city of Marburg, Germany. They have different ethnical backgrounds, three of them have European origin, two have Latin American origin, and one has Asian origin. The experimental procedure followed the tenets of the Declaration of Helsinki and was approved by the local ethics committee of the Psychology Department at Marburg University proposal number 2025-78 k).

### Apparatus

#### Relation experiment

A five-primary Maxwellian-view photostimulator system was used to record the pupillary light reflex (PLR). The system was previously described in the literature^33^. It consisted of five light-emitting diodes (LEDs) of different wavelengths and narrow-band interference filters (Peak wavelength [FWHM]: 460 nm [12 nm]-blue, 488 nm [13 nm]-cyan, 544 nm [13 nm]-green, 592 nm [20 nm]-amber, 632 nm [22 nm]-red). A five-fiber-optics bundle was used to transport each LED light to a homogenizer, where the lights were combined. The output light (stimulus) was imaged on the observer’s pupil using a field lens and a 2 mm artificial pupil, resulting in a Maxwellian view. Through the artificial pupil, the observer saw a ring-shaped illuminated patch. Observers must fixate on the ring’s center. The PLR recording was consensual using an eye tracker (Cambridge Research System Ltd, Rochester, UK) at a sampling rate of 250 Hz. Scattering measurements were done using a commercial device (C-Quant; Oculus, Lynnwood, USA). This system is based on the psychophysical method of “compensation comparison”, explained in detail elsewhere^74^. Ocular transmittance measurements were performed using the commercial OQAS double-pass system (Visiometrics SL, Terrassa, Spain) following the methodology described by Sánchez and colleagues^75^.

#### Simulation experiment

We used a Newtonian view setup. An ad-hoc tetra-chromatic projector (VPixx Technologies, Saint-Bruno, QC, Canada) was used to project an image into a rear projection screen (Stewart Filmscreen, Torrance, CA, USA). Using an eye-tracker (Eyelink 1000+, SR Research Ltd. Ontario, Canada) and the Eyelink toolbox^76^, we monitored the gaze position and pupil diameter of the observer. The simulation filters that we used were a Black Pro-Mist 2 filter (BPM 2; The Tiffen Company, Hauppauge, NY, USA) that mimics an early cataract^41–43^, a neutral density filter (LEE Filters, Burbank, CA, USA) with a transmittance of 64% was used to simulate a reduction in transmittance, and a yellowish filter (765, LEE Filters, Burbank, CA, USA) was used to simulate a yellowing in the pre-receptoral media. This filter used by a 20 years old participant mimics an eye between 60 to 80 years old (Fig. S1C).

### Stimuli

#### Relation experiment

The fPLR paradigm was implemented using a stimulus generated with the silent substitution technique^32^. With this technique, it is possible to selectively stimulate each of the five types of photoreceptors in the human retina (S cones, M cones, L cones, rods, and melanopsin-containing ipRGCs) when implemented in the five-primary system. The technique of silent substitution, considering the primaries’ power distribution and the photoreceptor sensitivities, generates a stimulus that changes the excitation of one or more photoreceptor classes while keeping the excitation of the others constant (silenced). Therefore, it is possible to isolate a photoreceptor-driven PLR from that of the others. More details about the method of silent substitution and primary systems can be found in the literature^68,77–79^. Sinusoidal stimuli at 1 Hz and 40 s in duration were generated using the silent substitution method to isolate the pupil responses of S-cones (contrast = 30%), L+M-cones (contrast = 20%), and melanopsin-containing ipRGCs (contrast = 17%). In this way, we obtained the flickering pupil light response (fPLR) paradigm. The mean stimulus light level (10° standard observer) was 2000 td (636 cd/m^2^, 15.2 log quanta/cm^2^/s). The stimulus was a ring-shaped patch (outer border: 24°, inner border: 6°). The central part was covered by an occlusion patch to minimize the effect of the macular pigmentation.

#### Simulation experiment

Pupil responses were assessed consensually using blue and red light-pulse stimuli of 1 s duration. The blue light level (10° standard observer) was 10.8 cd/m^2^, while the red light level (10° standard observer) was 27 cd/m^2^, they were matched in irradiance to obtain 13.5 log quanta/cm^2^/s. The colored stimulus was followed by a dark screen that was present during six seconds. The stimuli were presented in a dark background. With this stimulation we obtained the pPLR. The stimulus was a ring-shaped patch (outer border: 10°, 15°, 20°, and 25°, inner border: 5°). The central part was turned off to minimize the effect of the macular pigmentation. The stimulus was modified by the intrusion of the simulation filters in transmittance, spectra and diffusion.

### Variables

#### Relation experiment

The independent variables were the intraocular scattering measured as the logarithm of straylight (logS), and the intraocular transmittance measured as the ocular transmittance index (log OTI). The dependent variables were those obtained from the fPLR. The isolated photoreceptor PLR parameters were the amplitude (mm) and phase (degrees) at 1 Hz, calculated in the frequency domain using the Fourier transform of the fPLR data.

#### Simulation experiment

The independent variables were the intraocular scattering, the intraocular transmittance, and the lens yellowing. Changes from the baseline were obtained by using filters that simulate a significant increment in those variables. The dependent variables of the pPLR calculated in the time domain were: 1) maximum pupil constriction (MC), defined as the minimum pupil diameter achieved at the stimulus onset and expressed in percentage value relative to the pupil baseline; 2) time to constriction peak (T2P; msec), defined as the time period from stimulus onset and the maximum constriction; and 3) the post-illumination pupil response (PIPR), which was the pupil diameters averaged from the second 5 to 6 after stimulus offset and was expressed in percentage value relative to the pupil baseline.

### Procedure

#### Relation experiment

Transmittance test: The measurements were taken in a dark room with the lights of the lab turned off after the subject was adapted to the darkness with the purpose of obtaining the largest possible natural pupil, while the exit pupil was controlled by the artificial diaphragm of the system, set to 4 mm. In each record, six double-pass images and the corresponding background were taken per subject (each obtained with an exposure time of 250 ms), after correcting spherical refractive errors with the Badal of the instrument. No glasses or contact lenses were used for the optical correction during measurements. From each set of six images, an average image was calculated and then the background was subtracted from this average. Finally, a cropped version of this double-pass image (256 × 256 pixels) was used for the analysis. The region of interest used for the analysis was a ring between 25 to 35 min of arc, whose center was in the centroid of the spot of the cropped double-pass image. To determine the transmittance of the ocular media, a series of double-pass images were recorded, each one was taken at different intensity of the laser diode, which was controlled by the power supply of the laser. The irradiance in the pupil plane was measured with a detector (E2V, Spindler & Hoyer). For all the measurements, the exposure levels were never greater than the maximum permissible exposure (14.45 W/m^2^), which was established by the current standard regulating the use of laser radiation in living tissue^80^. The gray level in the region of interest increased as the intensity of the laser increased. The average of the scattering computed as a mean gray level (called double-pass scattering (*DPS*)) varies linearly with the irradiance in the pupil plane. We have used the slope of the *DPS*/irradiance line to derive an ocular transmittance index (*OTI*). The OTI was related to the transmittance value in a quadratic fashion^75^. The measurements were taken at 780 nm. The session lasted 30 min approximately, including enough resting time between runs.

Scattering test: The measurements were performed in a dark laboratory room. Participants underwent 3 monocular test runs in one session. The session lasted 15 min approximately, including enough resting time between runs. The average of the logS parameter from the 3 repetitions was considered for the analysis.

Pupillometry test: The heterochromatic flicker photometry (HFP) test was conducted to obtain the primary setting ratios Blue/Cyan, Green/Cyan, Amber/Cyan, and Red/Cyan for individual calibrations. The HFP was repeated twice, and the average values of the four ratios were used to modify the primary lights presentation for each participant in the fPLR paradigm.

After individual calibration, the participants were dark-adapted for 10 min. The pupillary recordings were organized in stimulus blocks. Each block comprised a series of five consecutive stimuli of the same characteristics presented with an inter-stimulus interval (ISI) of 30 s. The silent substitution paradigm consisted of three photoreceptor blocks: S-cones, L+M-cones, and melanopsin; presented in random order. A light-adapting background (equal to the mean stimulus light level) was presented for 2 min at the beginning of each block and during the ISIs. Participants were instructed to keep their eyes open during the stimulus presentation and rest during the ISI. The pupil recording protocol and the HFP were completed in one session, lasting 45 min approximately.

Participants conducted the transmittance, scattering, and pupillometry tests in a randomized order. The HFP test was always performed before the pupillometry test, since their results were used for correcting individual variability in spectral sensitivity for the fPLR paradigm. In total, each participant conducted one hour and 30 min of tests, split into two sessions for the relation experiment.

#### Simulation experiment

The pupillary measurements were done consensually using an ad-hoc visual blocker that allowed vision of the screen by the right eye, while the left eye could not see the screen but was recorded by the camera and illuminated by the infrared LEDs of the eye tracker. At the start of each session, the participants conducted a gaze calibration with their naked eye. Then, they conducted 16 experimental conditions [two stimuli color (blue and red) by four filtering conditions (naked eye, scattering filter, transmittance filter, and yellowish filter)]. These conditions were randomized except for the stimulus color that was interleaved. Each condition was repeated at least three times. One trial consisted of a preparation screen (1 s.), a fixation cross on a dark screen (1 s.), the stimulus (1 s.), and a dark screen to measure the PIPR (6 s.). Participants were instructed not to blink and maintain fixation during the trial presentation. The ISI lasted at least five seconds and consisted of a noisy grayscale screen with a message indicating that the participant was able to blink and relax fixation during this period. Through a gaze-contingent paradigm^81^, we ensured that the participants could perceive the stimulus foveally. Using this paradigm, we discarded trials where the participants moved their gaze more than 1 deg away from the fixation cross, therefore controlling that the stimulus was presented at the required eccentricity.

### Statistics

Statistical analyses of our data were performed using Minitab (Minitab Inc.), Matlab (Mathworks Inc.) and Prism (GraphPad Software LLC) software. A repeated-measures ANOVA was conducted to account for the effects of stimulus color, filter, and size in the simulation experiment, followed by a post-hoc analysis using Tukey’s method for pairwise comparisons. We performed paired t-tests to compare pairwise differences in Figs. 2, 4, 6 and 7. Scatter plots to compare pairwise differences were made removing outliers and using the method provided Schütz and Gegenfurtner^82^. The significance level was set at 5% in all analyses. Simple linear regression analyses were performed for the relation experiment (Figs. 3 and 5).

## Supplementary Information

Below is the link to the electronic supplementary material.

Supplementary Material 1

## Data Availability

Pupillary and intraocular media data available at the following URL: 10.6084/m9.figshare.31795339

## References

[1] Gamlin, P. D. R. The pretectum: Connections and oculomotor-related roles. In *Progress in Brain Research* Vol. 151 (ed. Büttner-Ennever, J. A.) 379–405 (Elsevier, 2006). 10.1016/S0079-6123(05)51012-4 16221595

[2] Boettner, E. A. & Wolter, J. R. Transmission of the ocular media. *Invest. Ophthalmol. Vis. Sci.* **1**, 776–783 (1962).

[3] Paz Filgueira, C., Sánchez, R. F., Issolio, L. A. & Colombo, E. M. Straylight and visual quality on early nuclear and posterior subcapsular cataracts. *Curr. Eye Res.* **41**, 1209–1215 (2016). 26766561 10.3109/02713683.2015.1101139

[4] de Waard, P. W., IJspeert, J. K., van den Berg, T. J. & de Jong, P. T. Intraocular light scattering in age-related cataracts. *Invest. Ophthalmol. Vis. Sci.* **33**, 618–625 (1992). 1544787

[5] IJspeert, J. K., de Waard, P. W., van den Berg, T. J. & de Jong, P. T. The intraocular straylight function in 129 healthy volunteers; dependence on angle, age and pigmentation. *Vision Res.* **30**, 699–707 (1990). 2378063 10.1016/0042-6989(90)90096-4

[6] Karas, F. I. et al. Intraocular light scatter in eyes with the Boston type 1 keratoprosthesis. *Cornea* **38**, 50–53 (2019). 30157051 10.1097/ICO.0000000000001724

[7] Sharma, S. et al. Factors influencing the pupillary light reflex in healthy individuals. *Graefes. Arch. Clin. Exp. Ophthalmol.* **254**, 1353–1359 (2016). 26968720 10.1007/s00417-016-3311-4

[8] Rukmini, A. V., Milea, D., Aung, T. & Gooley, J. J. Pupillary responses to short-wavelength light are preserved in aging. *Sci. Rep.* **7**, 43832 (2017). 28266650 10.1038/srep43832 PMC5339857

[9] Silva, B., Sfer, A., Villar, M. A. D., Issolio, L. A. & Colombo, E. M. Pupil dynamics with periodic flashes: Effect of age on mesopic adaptation. *J. Opt. Soc. Am. A Opt. Image Sci. Vis.* **33**, 1546–1552 (2016). 27505653 10.1364/JOSAA.33.001546

[10] Norren, D. V. & Vos, J. J. Spectral transmission of the human ocular media. *Vision Res.* **14**, 1237–1244 (1974). 4428632 10.1016/0042-6989(74)90222-3

[11] Rieke, F. & Rudd, M. E. The challenges natural images pose for visual adaptation. *Neuron.* **64**, 605–616 (2009). 10.1016/j.neuron.2009.11.028 20005818

[12] Wang, Y. V. & Demb, J. B. Postreceptoral Mechanisms for Adaptation in the Retina. in The New Visual Neurosciences (eds Werner, J. S. & Chalupa, L. M.) (The MIT Press, Cambridge, MA, 2014).

[13] Delahunt, P. B., Webster, M. A., Ma, L. & Werner, J. S. Long-term renormalization of chromatic mechanisms following cataract surgery. *Visual Neuroscience.* **21**, 301–307 (2004). 10.1017/S0952523804213025 PMC2633455 15518204

[14] Barrionuevo, P. A., Colombo, E. M. & Issolio, L. A. Retinal mesopic adaptation model for brightness perception under transient glare. *J. Opt. Soc. Am. A.* **30**, 1236–1247 (2013). 10.1364/JOSAA.30.001236 24323111

[15] Eto, T. et al. Crystalline lens transmittance spectra and pupil sizes as factors affecting light-induced melatonin suppression in children and adults. *Ophthalmic Physiol. Opt.* **41**, 900–910 (2021). 33772847 10.1111/opo.12809

[16] Barrionuevo, P. A., Issolio, L. A. & Tripolone, C. Photoreceptor contributions to the human pupil light reflex. *J. Photochem. Photobiol.* **15**, 100178 (2023).

[17] Alpern, M. & Campbell, F. W. The spectral sensitivity of the consensual light reflex. *J. Physiol.* **164**, 478–507 (1962). 14012269 10.1113/jphysiol.1962.sp007033 PMC1359246

[18] Barrionuevo, P. A. et al. Assessing rod, cone, and melanopsin contributions to human pupil flicker responses. *IOVS* **55**, 719–727 (2014). 10.1167/iovs.13-13252 PMC3915766 24408974

[19] McDougal, D. H. & Gamlin, P. D. The influence of intrinsically photosensitive retinal ganglion cells on the spectral sensitivity and response dynamics of the human pupillary light reflex. *Vision. Res.* **50**, 72–87 (2010). 19850061 10.1016/j.visres.2009.10.012 PMC2795133

[20] Adhikari, P., Zele, A. J. & Feigl, B. The post-illumination pupil response (PIPR). *Invest. Opthalmol. Vis. Sci.* **56**, 3838 (2015). 10.1167/iovs.14-16233 26066752

[21] Spitschan, M., Jain, S., Brainard, D. H. & Aguirre, G. K. Opponent melanopsin and S-cone signals in the human pupillary light response. *Proc. Natl. Acad. Sci. U. S. A.* **111**, 15568–15572 (2014). 25313040 10.1073/pnas.1400942111 PMC4217411

[22] Barrionuevo, P. A. & Cao, D. Luminance and chromatic signals interact differently with Melanopsin activation to control the pupil light response. *J. Vis.* **16**, 29 (2016). 27690169 10.1167/16.11.29 PMC5054726

[23] Zele, A. J., Adhikari, P., Cao, D. & Feigl, B. Melanopsin and cone photoreceptor inputs to the afferent pupil light response. *Front. Neurol.*10.3389/fneur.2019.00529 (2019). 31191431 10.3389/fneur.2019.00529 PMC6540681

[24] Kimura, E. & Young, R. S. L. S-cone contribution to pupillary responses evoked by chromatic flash offset. *Vision Res.* **39**, 1189–1197 (1999). 10343835 10.1016/s0042-6989(98)00154-0

[25] Dacey, D. M. et al. Melanopsin-expressing ganglion cells in primate retina signal colour and irradiance and project to the LGN. *Nature* **433**, 749–754 (2005). 15716953 10.1038/nature03387

[26] Zele, A. J., Adhikari, P., Cao, D. & Feigl, B. Melanopsin driven enhancement of cone-mediated visual processing. *Vision Res.* **160**, 72–81 (2019). 31078661 10.1016/j.visres.2019.04.009

[27] Curcio, C. A., Sloan, K. R., Kalina, R. E. & Hendrickson, A. E. Human photoreceptor topography. *J. Comp. Neurol.* **292**, 497–523 (1990). 2324310 10.1002/cne.902920402

[28] Liao, H.-W. et al. Melanopsin-expressing ganglion cells on macaque and human retinas form two morphologically distinct populations. *J. Comp. Neurol.* **524**, 2845–2872 (2016). 26972791 10.1002/cne.23995 PMC4970949

[29] Nasir-Ahmad, S., Lee, S. C. S., Martin, P. R. & Grünert, U. Melanopsin-expressing ganglion cells in human retina: Morphology, distribution, and synaptic connections. *J. Comp. Neurol.* **527**, 312–327 (2019). 28097654 10.1002/cne.24176

[30] Loewenfeld, I. E. & Lowenstein, O. *The Pupil: Anatomy, Physiology, and Clinical Applications* (Iowa State University Press, 1993).

[31] Winn, B., Whitaker, D., Elliott, D. B. & Phillips, N. J. Factors affecting light-adapted pupil size in normal human subjects. *Invest. Ophthalmol. Vis. Sci.* **35**, 1132–1137 (1994). 8125724

[32] Estévez, O. & Spekreijse, H. The ‘silent substitution’ method in visual research. *Vision Res.* **22**, 681–691 (1982). 7112962 10.1016/0042-6989(82)90104-3

[33] Cao, D., Nicandro, N. & Barrionuevo, P. A. A five-primary photostimulator suitable for studying intrinsically photosensitive retinal ganglion cell functions in humans. *J. Vis.* **15**, 27 (2015). 25624466 10.1167/15.1.27 PMC4528566

[34] Woelders, T. et al. Melanopsin- and L-cone–induced pupil constriction is inhibited by S- and M-cones in humans. *PNAS* **115**, 792–797 (2018). 29311335 10.1073/pnas.1716281115 PMC5789936

[35] Rukmini, A. V., Milea, D. & Gooley, J. J. Chromatic pupillometry methods for assessing photoreceptor health in retinal and optic nerve diseases. *Front. Neurol.*10.3389/fneur.2019.00076 (2019). 30809186 10.3389/fneur.2019.00076 PMC6379484

[36] Kelbsch, C. et al. Standards in pupillography. *Front. Neurol.*10.3389/fneur.2019.00129 (2019). 30853933 10.3389/fneur.2019.00129 PMC6395400

[37] Verdon, W. & Howarth, P. A. The pupil’s response to short wavelength cone stimulation. *Vision Res.* **28**, 1119–1128 (1988). 3257014 10.1016/0042-6989(88)90138-1

[38] Kardon, R. et al. Chromatic pupil responses: Preferential activation of the melanopsin-mediated versus outer photoreceptor-mediated pupil light reflex. *Ophthalmology* **116**, 1564–1573 (2009). 19501408 10.1016/j.ophtha.2009.02.007

[39] Park, J. C. et al. Toward a clinical protocol for assessing rod, cone, and melanopsin contributions to the human pupil response. *IOVS* **52**, 6624–6635 (2011). 10.1167/iovs.11-7586 PMC3175993 21743008

[40] Park, J. C. & McAnany, J. J. Effect of stimulus size and luminance on the rod-, cone-, and melanopsin-mediated pupillary light reflex. *J. Vis.* **15**, 13 (2015). 25788707 10.1167/15.3.13 PMC4365915

[41] Barrionuevo, P. A., Colombo, E. M., Corregidor, D., Jaen, M. & Issolio, L. A. Evaluation of the intraocular scattering through brightness reduction by glare using external diffusers to simulate cataracts. *Opt. Appl.* **40**, 63–75 (2010).

[42] de Wit, G. C., Franssen, L., Coppens, J. E. & van den Berg, T. J. T. P. Simulating the straylight effects of cataracts. *J Cataract Refract Surg* **32**, 294–300 (2006). 16565008 10.1016/j.jcrs.2006.01.048

[43] Barrionuevo, P. A., Colombo, E. M., Vilaseca, M., Pujol, J. & Issolio, L. A. Comparison between an objective and a psychophysical method for the evaluation of intraocular light scattering. *J. Opt. Soc. Am. A* **29**, 1293–1299 (2012). 10.1364/JOSAA.29.001293 22751395

[44] McAdams, H. et al. Selective amplification of ipRGC signals accounts for interictal photophobia in migraine. *Proc. Natl. Acad. Sci.* **117**, 17320–17329 (2020). 32632006 10.1073/pnas.2007402117 PMC7382295

[45] Kelbsch, C. et al. Pupillary responses driven by ipRGCs and classical photoreceptors are impaired in glaucoma. *Graefes Arch. Clin. Exp. Ophthalmol.*10.1007/s00417-016-3351-9 (2016). 27099948 10.1007/s00417-016-3351-9

[46] Hu, X., Hisakata, R. & Kaneko, H. Effects of stimulus size, eccentricity, luminance, and attention on pupillary light response examined by concentric stimulus. *Vision. Res.* **170**, 35–45 (2020). 32244112 10.1016/j.visres.2020.03.008

[47] van den Berg, T. J. T. P. Intraocular light scatter, reflections, fluorescence and absorption: What we see in the slit lamp. *Ophthalmic Physiol. Opt.* **38**, 6–25 (2018). 29265476 10.1111/opo.12426

[48] Cox, A. J., DeWeerd, A. J. & Linden, J. An experiment to measure Mie and Rayleigh total scattering cross sections. *Am. J. Phys.* **70**, 620–625 (2002).

[49] Castro-Torres, J. J., Martino, F., Casares-López, M., Ortiz-Peregrina, S. & Ortiz, C. Visual performance after the deterioration of retinal image quality: Induced forward scattering using Bangerter foils and fog filters. *Biomed. Opt. Express. BOE.* **12**, 2902–2918 (2021). 34123509 10.1364/BOE.424715 PMC8176796

[50] Łabuz, G. et al. Validation of a spectral light scattering method to differentiate large from small particles in intraocular lenses. *Biomed. Opt. Express. BOE.* **8**, 1889–1894 (2017). 28663871 10.1364/BOE.8.001889 PMC5480586

[51] Lowenstein, O., Kawabata, H. & Loewenfeld, I. E. The pupil as indicator of retinal activity. *Am. J. Ophthalmol.* **57**, 569–596 (1964). 14139299 10.1016/0002-9394(64)92504-8

[52] Alpern, M. Relation of visual latency to intensity. *AMA Arch. Ophthalmol.* **51**, 369–374 (1954). 13123622 10.1001/archopht.1954.00920040379011

[53] Link, N. & Stark, L. Latency of the pupillary response. *IEEE Trans. Biomed. Eng.* **35**, 214–218 (1988). 3350551 10.1109/10.1365

[54] Bremner, F. D. Pupillometric evaluation of the dynamics of the pupillary response to a brief light stimulus in healthy subjects. *Invest. Ophthalmol. Vis. Sci.* **53**, 7343–7347 (2012). 23036998 10.1167/iovs.12-10881

[55] Ellis, C. J. The pupillary light reflex in normal subjects. *Br. J. Ophthalmol.* **65**, 754–759 (1981). 7326222 10.1136/bjo.65.11.754 PMC1039657

[56] Eto, T. & Higuchi, S. Review on age-related differences in non-visual effects of light: Melatonin suppression, circadian phase shift and pupillary light reflex in children to older adults. *J. Physiol. Anthropol.* **42**, 11 (2023). 37355647 10.1186/s40101-023-00328-1 PMC10290329

[57] Charman, W. N. Age, lens transmittance, and the possible effects of light on melatonin suppression. *Ophthalmic Physiol. Opt.* **23**, 181–187 (2003). 12641706 10.1046/j.1475-1313.2003.00105.x

[58] Lazar, R., Degen, J., Fiechter, A.-S., Monticelli, A. & Spitschan, M. Regulation of pupil size in natural vision across the human lifespan. *R. Soc. Open Sci.* **11**, 191613 (2024). 39100191 10.1098/rsos.191613 PMC11295891

[59] Adhikari, P., Pearson, C. A., Anderson, A. M., Zele, A. J. & Feigl, B. Effect of age and refractive error on the melanopsin mediated post-illumination pupil response (PIPR). *Sci. Rep.* **5**, 17610 (2015). 26620343 10.1038/srep17610 PMC4664956

[60] Herbst, K. et al. Intrinsically photosensitive retinal ganglion cell function in relation to age: A pupillometric study in humans with special reference to the age-related optic properties of the lens. *BMC Ophthalmol.* **12**, 4 (2012). 22471313 10.1186/1471-2415-12-4 PMC3411473

[61] Ba-Ali, S., Brøndsted, A. E., Andersen, H. U., Jennum, P. & Lund-Andersen, H. Pupillary light responses in type 1 and type 2 diabetics with and without retinopathy. *Acta Ophthalmol.* **98**, 477–484 (2020). 31943805 10.1111/aos.14348

[62] Park, J. C. et al. Pupillary responses in non-proliferative diabetic retinopathy. *Sci. Rep.* **7**, srep44987 (2017). 10.1038/srep44987 PMC5362954 28332564

[63] Dumpala, S., Zele, A. J. & Feigl, B. Outer retinal structure and function deficits contribute to circadian disruption in patients with type 2 diabetes. *Invest. Ophthalmol. Vis. Sci.* **60**, 1870–1878 (2019). 31042793 10.1167/iovs.18-26297

[64] Kankipati, L., Girkin, C. A. & Gamlin, P. D. The post-illumination pupil response is reduced in glaucoma patients. *Invest. Ophthalmol. Vis. Sci.* **52**, 2287 (2011). 21212172 10.1167/iovs.10-6023 PMC3080733

[65] Feigl, B., Mattes, D., Thomas, R. & Zele, A. J. Intrinsically photosensitive (melanopsin) retinal ganglion cell function in glaucoma. *Invest. Ophthalmol. Vis. Sci.* **52**, 4362 (2011). 21498620 10.1167/iovs.10-7069

[66] McAdams, H., Igdalova, A., Spitschan, M., Brainard, D. H. & Aguirre, G. K. Pulses of melanopsin-directed contrast produce highly reproducible pupil responses that are insensitive to a change in background radiance. *Invest. Ophthalmol. Vis. Sci.* **59**, 5615–5626 (2018). 30481278 10.1167/iovs.18-25219 PMC6262648

[67] Nugent, T. W. & Zele, A. J. A five-primary Maxwellian-view display for independent control of melanopsin, rhodopsin, and three-cone opsins on a fine spatial scale. *J. Vis.* **22**, 20 (2022). 36445714 10.1167/jov.22.12.20 PMC9716233

[68] Barrionuevo, P. A., Preciado, O. U., Sandoval Salinas, M. L. & Issolio, L. A. Optical stimulation systems for studying human vision. In *Progress in Brain Research* Vol. 273 (eds Santhi, N. & Spitschan, M.) 13–32 (Elsevier, 2022). 10.1016/bs.pbr.2022.04.003 35940713

[69] Tsujimura, S., Ukai, K., Ohama, D., Nuruki, A. & Yunokuchi, K. Contribution of human melanopsin retinal ganglion cells to steady-state pupil responses. *Proc. Biol. Sci.* **277**, 2485–2492 (2010). 20375057 10.1098/rspb.2010.0330 PMC2894926

[70] Murray, I. J., Kremers, J., McKeefry, D. & Parry, N. R. A. Paradoxical pupil responses to isolated M-cone increments. *J. Opt. Soc. Am. A. JOSAA.* **35**, B66–B71 (2018). 10.1364/JOSAA.35.000B66 29603924

[71] Coppens, J. E., Franssen, L. & van den Berg, T. J. T. P. Wavelength dependence of intraocular straylight. *Exp. Eye Res.* **82**, 688–692 (2006). 16293245 10.1016/j.exer.2005.09.007

[72] Joyce, D. S., Feigl, B. & Zele, A. J. Melanopsin-mediated post-illumination pupil response in the peripheral retina. *J. Vis.* **16**, 5 (2016). 27271992 10.1167/16.8.5

[73] Adhikari, P., Feigl, B. & Zele, A. J. Rhodopsin and melanopsin contributions to the early redilation phase of the post-illumination pupil response (PIPR). *PLoS ONE* **11**, e0161175 (2016). 27548480 10.1371/journal.pone.0161175 PMC4993463

[74] Franssen, L., Coppens, J. E. & van den Berg, T. J. T. P. Compensation comparison method for assessment of retinal straylight. *Invest. Ophthalmol. Vis. Sci.* **47**, 768–776 (2006). 16431978 10.1167/iovs.05-0690

[75] Sánchez, R. et al. Transmittance measurement of the in vivo human eye with a double-pass system. *Opt. Appl.* **51**, 1–21 (2021).

[76] Cornelissen, F. W., Peters, E. M. & Palmer, J. The Eyelink Toolbox: Eye tracking with MATLAB and the Psychophysics Toolbox. *Behav. Res. Methods Instrum. Comput.* **34**, 613–617 (2002). 12564564 10.3758/bf03195489

[77] Nugent, T. W. et al. Protocol for isolation of melanopsin and rhodopsin in the human eye using silent substitution. *STAR Protocols* **4**, 102126 (2023). 36892996 10.1016/j.xpro.2023.102126 PMC10011832

[78] Barrionuevo, P. A., Sandoval Salinas, M. L. & Fanchini, J. M. Are ipRGCs involved in human color vision? Hints from physiology, psychophysics, and natural image statistics. *Vision Res.* **217**, 108378 (2024). 38458004 10.1016/j.visres.2024.108378

[79] Spitschan, M. & Woelders, T. The method of silent substitution for examining melanopsin contributions to pupil control. *Front. Neurol.*10.3389/fneur.2018.00941 (2018). 30538662 10.3389/fneur.2018.00941 PMC6277556

[80] IEC 60825-1 Standard. *Safety of Laser Products*. (International Electrotechnical Commission, Geneva, 2007).

[81] Barrionuevo, P. A., Schütz, A. C. & Gegenfurtner, K. R. Increased brightness assimilation in rod vision. *iScience*10.1016/j.isci.2024.111609 (2025). 39898055 10.1016/j.isci.2024.111609 PMC11787610

[82] Schütz, A. C. & Gegenfurtner, K. R. Within-subject confidence intervals for pairwise differences in scatter plots. *Psychon. Bull. Rev.* **32**, 3238–3251 (2025). 40877721 10.3758/s13423-025-02750-1 PMC7618193

