# Supplementary material for: Simulated and natural changes in intraocular transparency selectively affect photoreceptor-specific pupil responses
Source: Sci Rep. 2026 May 29;16:26013. doi: 10.1038/s41598-026-54502-3 (PMC13486483; doi:10.1038/s41598-026-54502-3)
Supplement: Supplementary file 1 [file 41598_2026_54502_MOESM1_ESM.docx]

Supplementary Information

**Simulated and natural changes in intraocular transparency selectively affect photoreceptor-specific pupil responses**

Constanza Tripolone, Roberto Sánchez, Luis A. Issolio, Pablo A. Barrionuevo

**Fig. S1**. **The effect of the external filters.** A) The effect of the filter BPM2 on the level of scattering of an artificial eye using double-pass images. The spectral transmittance of the BPM2 filter is in the inset. B) The spectral profile of the foil neutral density (ND) filter that we used to reduce the transmittance level. In the same panel, the normalized sensitivities of luminance (L+M), S-cones, and melanopsin are included. C) The transmittance effect of the yellow filter to a lens of a 20-year-old observer is shown. In the same panel, the effect of age on the natural lens spectral transmittance is shown for comparison purposes (data from CIE 170-1:2006). Relative difference of the excitations produced by the external filters of scattering (D), transmittance (E), and yellowing (F).

**Fig. S2**. **Subtracting red PIPR from the blue PIPR.** The difference between the blue PIPR and the red PIPR (dPIPR) for the scattering experiment. Panels contain the dPIPR values of each participant (blue dots) for each stimulus size: 10° (A), 15° (B), 20° (C), and 25° (D). The black dots represent the mean values, the vertical and horizontal thin lines represent the stand-alone effect confidence interval, and the diagonal wide line represents the confidence interval for the pairwise differences. Note that neither of the panels contains significant pairwise differences.

**Fig. S3**. **The relation between latency, velocity, and T2P differences (Δ) between naked eye and eye + filter.** The ΔT2P was not correlated with Δlatency (A) and Δvelocity (B) for the scattering filter, nor was it for the transmittance filter (C and D). The latency was computed as the time interval between the light onset and pupil constriction onset. The velocity was computed as the maximum value of the first derivative of the pupil response in the interval 0 to 1500 ms. Data points represent the average across participants for the two colors and the four sizes of the stimulation. We represent the mean pupil traces of the scattering experiment for the naked eye with the eye + diffuser filter for blue stimulation (E, I, M, and Q) and for red stimulation (F, J, N, and R). The mean pupil traces of the transmittance experiment for the naked eye with the eye + neutral density filter are also shown for blue stimulation (G, K, O, and S) and for red stimulation (H, L, P, and T). The vertical pointed line represents the T2P of the naked eye, while the vertical dashed line represents the T2P of the eye+filter. Note the consistently delayed response that the diffuser produces (two columns to the left). The shaded area around the mean pupil traces represents SEM.

**Table S1**. **Differences in iris pigmentation color**. Number of participants in the two experiments clasifyed by the iris pigmentation color.

| Experiment | green | blue/green | brown | dark brown |
| --- | --- | --- | --- | --- |
| Relation | 1 | 0 | 3 | 4 |
| Simulation | 0 | 1 | 3 | 3 |
